# The Anatomical Relationship Between the Hyoid Bone and the Carotid Arteries

**DOI:** 10.3390/diagnostics15121485

**Published:** 2025-06-11

**Authors:** Nektaria Karangeli, George Triantafyllou, Panagiotis Papadopoulos-Manolarakis, George Tsakotos, Katerina Vassiou, Marianna Vlychou, Panagiotis Papanagiotou, Maria Piagkou

**Affiliations:** 1 Department of Anatomy, School of Medicine, Faculty of Health Sciences, National and Kapodistrian University of Athens, 11 527 Athens, Greece; nekkarangeli@gmail.com (N.K.); georgerose406@gmail.com (G.T.); p.papado89@gmail.com (P.P.-M.); gtsakotos@gmail.com (G.T.); 2 Department of Neurosurgery, General Hospital of Nikaia-Piraeus, 18 454 Nikaia, Greece; 3 Department of Anatomy, Faculty of Medicine, University of Thessaly, 41 334 Larissa, Greece; avassiou@gmail.com; 4 Department of Radiology, University Hospital of Larissa, 41 334 Larissa, Greece; mvlychou@med.uth.gr; 5 Department of Radiology, Aretaieion University Hospital, School of Medicine, Faculty of Health Sciences, National and Kapodistrian University of Athens, 11 527 Athens, Greece; papanagiotou@me.com

**Keywords:** internal carotid artery, external carotid artery, common carotid artery, hyoid bone, anatomical relationship, topography, anatomy, variation

## Abstract

**Background**: The anatomical relationship between the carotid arteries (CAs) and the hyoid bone (HB) has significant clinical implications. The present study investigates the spatial relationship between the HB and common carotid artery (CCA), internal carotid artery (ICA), external carotid artery (ECA), and carotid bifurcation (CB), analyzing their morphological variants and topographical associations. **Materials and Methods**: Computed tomography angiographies (CTAs) from 100 patients (50 males, 50 females; mean age 62.2 ± 14.5 years) were analyzed. Measurements included the HB greater horn length (GHL) and greater horn angle (GHA), CA distances, and classification of relationships of the CAs with the HB. **Results**: The mean GHL was 27.72 ± 3.91 mm, and the mean GHA was 110.36 ± 6.06 degrees. Males exhibited significantly longer GHs than females (*p* < 0.001). Suprahyoid bifurcations occurred in 35.5% of cases, with a mean CCA-GH distance of 4.54 ± 2.67 mm. Infrahyoid bifurcations were identified in 64.5% of cases, with mean ECA-GH and ICA-GH distances of 4.10 ± 3.11 mm and 6.72 ± 3.85 mm, respectively. GHL significantly influenced these distances. Type 6 (ECA positioned laterally to the GH) was the most prevalent carotid–hyoid configuration (18.5%). **Conclusions**: The present study provides essential insights into the topographical variability of the CAs relative to the HB, offering valuable guidance for surgical planning and vascular risk assessment. These findings are crucial for procedures such as carotid endarterectomy, where the proximity of the carotid arteries to the hyoid bone impacts the risk of vascular injury. Further research is warranted to explore the clinical implications of these anatomical relationships.

## 1. Introduction

The head and neck complex vascular anatomy has undergone extensive examination due to its significant clinical importance, providing essential insights for precise surgical strategizing and intervention planning. Traditional references typically assert that the common carotid artery (CCA) bifurcates into the internal and external carotid arteries (ICA and ECA) at a location superior to the thyroid lamina, with the upper margin of the thyroid cartilage signifying the average carotid bifurcation (CB) level. Nevertheless, CB may occur at various vertebral levels, ranging as high as the hyoid bone (HB) or the styloid process (SP) or as low as the cricoid cartilage, indicating potential anatomical relationships (topography) with essential structures [1].

The carotid arteries’ (CAs) course has been the focus of extensive investigation, due to their crucial role in cerebrovascular perfusion and significant clinical implications in various pathological conditions and surgical interventions [2]. Although their branching patterns have been meticulously characterized, their topographical relationships with adjacent structures, such as the HB, have garnered comparatively limited attention. Elucidating potential correlations between the CAs and prominent anatomical landmarks is critical for enhancing the understanding of the intricate regional architecture [2,3,4,5,6,7,8].

The HB is a small, solitary structure located in the midline of the anterior neck, positioned between the base of the mandible superiorly and the fourth cervical vertebra inferiorly, with its location just superior to the thyroid cartilage. This bone has five distinct elements: a central body and two greater and lesser horns (GHs and LHs). Although the HB is not directly articulated with adjacent bones, it is intricately connected to a complex network of tendons and muscles. It is securely suspended within the anterior triangle of the neck by muscles that originate from the larynx, pharynx, tongue, and the floor of the mouth [1].

The potential topography (relationships) between the CCA, ICA, ECA, and the CB concerning the HB holds significant clinical relevance. Mechanical compression leading to atherosclerosis formation, transient ischemic attacks, and strokes, as well as aberrant courses that lead to complications during neck surgeries, are some of the clinical implications that are correlated with the relationships of these vital structures. However, there exists a paucity of studies investigating this vital topographical relationship, contrary to the vast majority of studies reporting the branching pattern [9,10]. The close spatial association of these vascular structures with the HB and the variability in their topographical anatomy have critical implications for preoperative planning and the precise execution of surgical procedures within this region. Notably, there is a lack of research addressing this proximity. Furthermore, this anatomical configuration may influence the emergence of pathological conditions such as atheromatosis, potentially exacerbated by the mechanical stress induced by their proximity. The present study systematically examines spatial relationships among the CCA, ICA, ECA, and CB concerning the HB while also analyzing variants and topographical associations.

## 2. Materials and Methods

**Figure 1:**
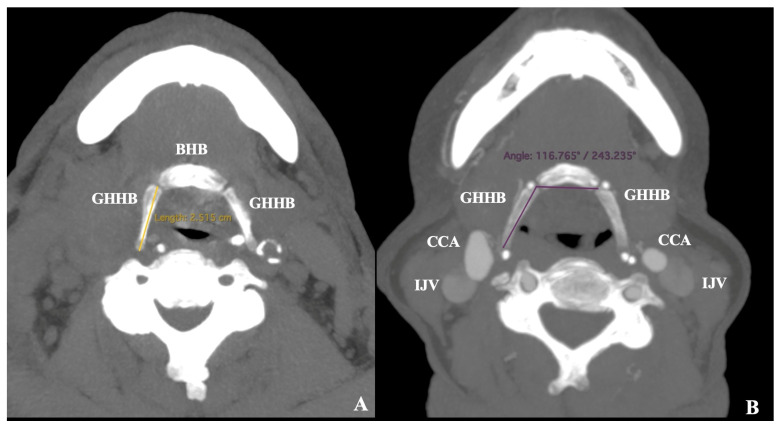
The measurements were obtained from the hyoid bone (HB). (**A**) Length of the greater horn (GHHB); (**B**) Angulation of the greater horn (GHHB). BHB: body of the hyoid bone, CCA: common carotid artery, IJV: internal jugular vein.

**Figure 2:**
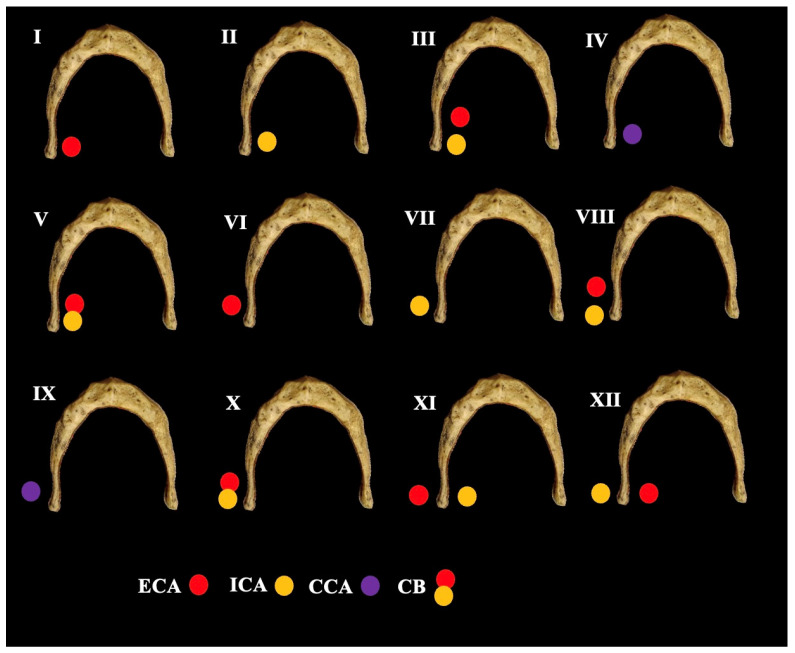
The topographical relationship between the hyoid bone and the carotid arteries (CCA: common carotid artery, ECA: external carotid artery, ICA: internal carotid artery, and CB: carotid bifurcation) is classified into types.

**Figure 3:**
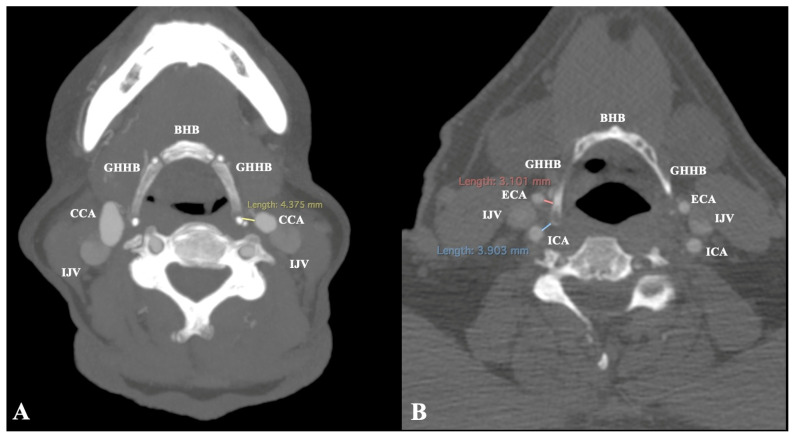
The measurements were obtained between the carotid arteries and the hyoid bone (HB). (**A**) Suprahyoid carotid bifurcation, common carotid artery (CCA) distance from the greater horn (GHHB). (**B**) Infrahyoid carotid bifurcation, external and internal carotid arteries (ECA and ICA) distance from the greater horn (GHHB). BHB: body of the hyoid bone, and IJV: internal jugular vein.

Following ethical approval, an archived collection of 100 computed tomography angiographies (CTAs) was obtained from the General Hospital of Nikaia Piraeus (number: 56485/13 November 2024). The current retrospective study included data obtained between December 2024 and February 2025. The CTAs were performed using a helical high-speed, low-dose scanner (SOMATOM go. Top, 128-slice configuration, Siemens, Munich, Germany) with the patient’s head in the supine neutral position, following the injection of 60 mL of a 30% iodine solution at a flow rate of 4–4.5 mL/s. The sample included 50 females and 50 males, with a mean age of 62.2 ± 14.5 years. The inclusion criteria required CTAs with at least 1 mm resolution per slice, adequate scan quality, and the absence of pathological processes that could distort arterial anatomy (carotid artery disease or prior neck surgery), consistent with previous studies [3]. The study was conducted and documented using the Horos Software version 3.3.6 (Horos Project, New York, NY, USA). Data was obtained through multiplanar reconstruction of axial, coronal, and sagittal slices and three-dimensional volume reconstruction. The following parameters of the HB were evaluated by two researchers (NK, GTr):

- GH length (GHL) is the maximum distance from the junction with the HB body (Figure 1)
- GH angle (GHA) is the angle between the bilateral junction of the GH with the HB body, up to the tip of the GH (Figure 1)

- The relationship between HB and the CAs was assessed according to Manta et al. (2023) [11] classification system (Figure 2)
- In cases of suprahyoid CB, the minimum distance GH-CCA was measured (Figure 3)
- In cases of infrahyoid CB, the minimum distances GH-ICA and GH-ECA were measured (Figure 3)

Statistical analysis was conducted using IBM SPSS Statistics for macOS, Version 29 (IBM Corp., Armonk, NY, USA). Nominal data from unpaired observations were compared using the Chi-square test, while McNemar’s test was used for paired observations. Normality was evaluated using the Shapiro–Wilk test. Continuous variables were analyzed according to their measurement types. Mean comparisons across more than two groups employed one-way ANOVA if the data conformed to a normal distribution; otherwise, the Kruskal–Wallis test was applied. Linear regression was conducted for continuous variables to examine the impact of the GHL and GHA on the CCA, ECA, and ICA distances. Results are presented as means and standard deviations unless otherwise noted. A *p*-value of less than 0.05 was considered statistically significant.

Intraclass Correlation Coefficient (ICC) was utilized to ensure reliability and accuracy in the process. The mean value of the two investigators’ measurements was calculated for continuous variables. For continuous data, the ICC was computed to evaluate consistency between raters, with a value of 0.75 representing good agreement.

## 3. Results

The mean value of GHL was 27.72 ± 3.91 mm, and the mean value of the GHA was 110.36 ± 6.06 degrees. The morphometric measurements according to side and sex are summarized in Table 1. Male patients had significantly longer GHs than females (*p* < 0.001). The ICC for the morphometric measurements was 0.89, indicating excellent reliability between investigators.

A suprahyoid CB was identified in 71 sides (35.5%). In these cases, the mean distance CCA-GH was 4.54 ± 2.67 mm. This distance was unaffected by sex (*p* = 0.268) and side (*p* = 0.824). An infrahyoid CB was observed in 129 sides (64.5%). In these cases, the mean distance ECA–GH was 4.10 ± 3.11 mm, and ICA–GH was 6.72 ± 3.85 mm. These distances were unaffected by side (*p* = 0.273 and *p* = 0.100) and sex (*p* = 0.491 and *p* = 0.816).

The distances were correlated with the GHL and GHA; the results are summarized in Table 2. In cases of infrahyoid CB, the ECA and ICA were negatively correlated with the GHL; therefore, when the GH was longer, the ECA and ICA were closer to the HB.

**Figure 4:**
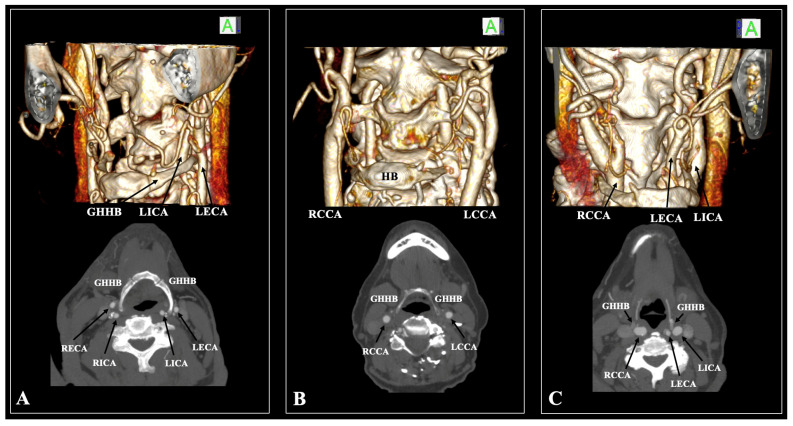
Examples of the cases included in the current study. (**A**) Three-dimensional reconstruction and axial slice of the 68-year-old male patient with type 11 (left side) and type 8 (right side); (**B**) Three-dimensional reconstruction and axial slice of the 83-year-old female patient with type 9 (left side) and type 0 (right side); (**C**) Three-dimensional reconstruction and axial slice of the 67-year-old female patient with type 12 (left side) and type 4 (right side).

The HB and the ICA–ECA relationship were assessed according to Manta et al.’s (2023) [11] classification system (Figure 4). The following results were obtained: Type 0—106 sides (53%), Type 1—1 side (0.5%), Type 2—0 sides (0%), Type 3—0 sides (0%), Type 4—1 side (0.5%), Type 5—0 sides (0%), Type 6—37 sides (18.5%), Type 7—0 sides (0%), Type 8—29 sides (14.5%), Type 9—14 sides (7%), Type 10—6 sides (3%), Type 11—5 sides (2.5%), and Type 12—1 side (0.5%). The types were not significantly different between sexes (*p* = 0.607) and sides (*p* = 0.840). It is essential to highlight that the distance GH–CCA was statistically significant between the various types (*p* = 0.037), as well as the distance CH–ECA (*p* < 0.001) and GH–ICA (*p* < 0.001).

## 4. Discussion

In the current cohort study, the CB was identified at a suprahyoid level at 35.5%, whereas an infrahyoid CB was observed at 64.5%. This predominance of infrahyoid CB aligns with established literature [12]. Furthermore, it was determined that the ECA exhibited the closest spatial relationship to the GH, followed by the CCA and, most distally, the ICA. Notably, in 53% of individuals, no discernible CA–HB relationship was identified. Additionally, types 2, 3, 5, and 7 were absent in the present cohort. The most frequently encountered anatomical configuration was type 6 (ECA positioned laterally to the GH), which was present in 18.5%. Types 1, 4, 10, 11, and 12 were not observed bilaterally. The specific carotid–hyoid configurations significantly influenced spatial relationships among the CCA, ECA, and ICA. Moreover, while variants in the GHA did not exert a measurable effect on the hyoid–arterial distances, the GHL was a critical determinant in modulating these spatial parameters.

It is crucial to recognize that the HB demonstrates highly varied morphological characteristics intrinsically connected to individual differences in sex, height, and body mass [13]. A growing body of literature has provided strong evidence supporting the idea that HB has sexually dimorphic traits [14]. Abdelkader et al. [13], using machine learning techniques to study the HB in an Egyptian group, showed that males have significantly larger HBs than females, with all dimensions statistically greater in males. On the other hand, Fakhry et al. [15] reported that the GHL was higher in males, while an increased GHA was identified in females. In the present study, GHL was greater in males; however, sex was not determined to affect the GHA significantly. This indicates a sex-based anatomical difference that may influence the spatial relationship between the carotid arteries and hyoid bone during carotid endarterectomy.

Numerous authors have sought to establish a classification framework for HB morphology, with prevailing taxonomies delineating five principal configurations: U-shaped, open, triangular, horseshoe-shaped, and trapezoidal [16,17,18,19]. Hernandez et al. conducted a comprehensive morphometric investigation into the multiple dimensions of HB variability, specifically assessing asymmetry, isometry, and isomorphy, thereby elucidating the extensive morphological plasticity of this structure [20]. These morphological variations’ profound clinical and forensic implications necessitate rigorous study [13]. However, it is essential to highlight that evidence-based anatomical research previously discouraged morphological classification using shapes [21].

CA variability represents a highly complex and thoroughly researched area, with a vast array of literature aimed at clarifying the CAs’ branching patterns [3]. The CB has been extensively examined due to its considerable variability and clinical relevance as a site prone to atherosclerotic plaque formation, primarily due to hemodynamic turbulence [22]. Additionally, CB topography has significant implications for diagnostic imaging, surgical guidance, and endovascular procedures such as carotid endarterectomy [22]. Beyond its direct clinical importance, CA morphology is a key focus in embryological research and genetic studies, providing insights into evolutionary adaptations within anthropological contexts.

The topographical relationship between the CAs and the HB remains a relatively underexplored area within vascular anatomy [11]. While several reports have suggested ischemic events possibly linked to this anatomical interaction, a thorough investigation is absent [23,24,25,26]. Furthermore, there is limited research on classifying the potential correlations between the CAs and the HB and analyzing their morphometric characteristics [11,27]. Although HB has been proposed to contribute to developing atherosclerotic lesions within the CAs, its definitive role remains unclear [11]. Additionally, few studies have addressed how variants in this anatomical relationship may influence parapharyngeal space surgical approaches [11]. This study aims to enhance scientific understanding of these critical aspects, thus contributing to the broader body of knowledge.

Manta et al. [11] systematically categorized 12 distinct variants in the spatial interrelation between the CAs and the GH to delineate the spectrum of carotid–hyoid topographical variability. Their findings revealed that in 57% of cases, no direct association between the CAs and the HB was observed. Consistent with our study, they identified type 6 as the most prevalent configuration. Furthermore, 104 of 147 analyzed cases exhibited bilateral symmetry in carotid–hyoid relationships [11].

Beyond anatomical classification, Manta et al. [11] highlighted the critical clinical implications of these interrelationships, particularly the potential for mechanical compression of the ICA, which may lead to adverse effects on cerebral circulation. They emphasized the need for increased surgical vigilance during cervical procedures involving the HB, as inadvertent vascular compromise may occur where the CAs are close to the hyoid framework.

Lemaire et al. [28] found no variant of the CB with the HB as a landmark. They observed that the CB was always located inferior and posterior to the tip of the HB’s GH.

However, several reports have documented diverse variants in carotid–hyoid topographical relationships, highlighting these configurations’ anatomical complexity and clinical significance [11]. The first documented case in 1999 described an 85-year-old patient presenting with a right hemispheric transient ischemic attack. Diagnostic evaluation revealed a 90% stenosis of the right ICA, with the GH’s concomitant compression and indentation of the vessel [29]. Kolbel et al. [30] reported a case of symptomatic stenosis of the right ICA, medially positioned relative to the HB. ICA entrapment was identified as the underlying cause of symptoms, necessitating resection of the right GH to restore vascular patency [25]. Three other cases of type 11 have been presented and were correlated with transient ischemic attack [24,31,32]. Kho et al. [32] presented a case of cryptogenic stroke caused by the laterally positioned ECA and ICA to the HB. Mori et al. [33] reported a case with the linguofacial trunk and the ICA lateral to the HB (type 7). Renard and Freitag [34] reported a type 8 case that coexisted with an elongated SP. Three more cases of type 8 have been reported, and a unique coexisted with type 10 [35,36,37]. A type 10 relationship was reported by Liu et al. [38], while a type 9 was reported by Schneider and Kortman [39].

Keshelava et al. [40] documented a case involving a patient who experienced recurrent transient ischemic attacks and a sudden loss of consciousness. CTA disclosed an 80% atherosclerotic stenosis of the ICA, accompanied by the ECA entrapment by the GH, which corresponds to type 12 in Manta’s classification system. The CB and the ICA were found to be in direct contact with the GH. Surgical exploration confirmed the presence of ECA compression, necessitating the resection of a 1 cm segment of the elongated GH to alleviate vascular entrapment and prevent the formation of an ECA pseudoaneurysm. This is the sole case report documenting the ECA entrapment.

Salaun-Penquer et al. [27] conducted a comprehensive retrospective analysis of CTAs involving patients who had undergone carotid endarterectomy for symptomatic or asymptomatic carotid stenosis. The primary objective of their study was to delineate the anatomical interplay between the HB and the CAs as portrayed by CTAs, with a particular focus on evaluating the hyoid–carotid distance, the positional relationship of the CAs relative to the HB, and variations in HB morphology. Furthermore, they explored potential correlations between these anatomical parameters and the severity of CA stenosis and stenosis-related cerebrovascular events. The authors classified the positional relationships of the CAs relative to the HB into four distinct categories: anterolateral, anteromedial, posterolateral, and posteromedial. Among these classifications, the posterolateral position was found to be the most frequent, occurring in 82% of cases, followed by the posteromedial position (9%), anterolateral position (8%), and anteromedial position (1%). Their findings indicated a significant association between the positioning of the CAs and the hyoid–carotid distance, with CAs positioned anteromedially demonstrating the shortest distances. At the same time, those in the posterolateral quadrant exhibited the greatest separation. Notably, no significant correlation was identified between the severity of CA stenosis and either hyoid–carotid distance or GHL, thereby highlighting the complexities inherent in the vascular and skeletal interactions within the cervical region [27].

Despite the extensive research on CB patterns, studies examining the topographical diversity of the CA pathways remain comparatively limited. A subset of investigations has concentrated on the spatial relationship between the SP and the CAs [3,4,5,6]. Calota et al. [6] conducted a detailed investigation into the course of the ECA concerning the SP, identifying significant variants in its trajectory. Triantafyllou et al. [4] further analyzed the implications of SP elongation and the variable ossification of the stylohyoid chain (SHC), emphasizing their potential influence on the spatial configuration of both the ICA and ECA. Another study investigated the effect of SP angulation on the trajectory of the CAs, highlighting its potential clinical consequences [5]. Our prior research specifically focused on the retrostyloid and retromandibular patterns of the ECA, underscoring the critical importance of these variations in surgical planning, particularly in reducing the risk of iatrogenic vascular injury during procedures involving the parapharyngeal space [3]. Dimitru et al. [8] highlighted a frequently overlooked phenomenon—the tortuosity of the retromandibular segment of the ECA—further emphasizing the necessity for comprehensive anatomical characterization to enhance surgical safety and procedural efficacy.

The HB has been implicated as a contributing factor in developing atherosclerotic lesions in the CAs. The cases reviewed suggest that chronic microtraumatic forces arising from an atypical relationship between the HB and the CAs may accelerate plaque formation and heighten the risk of artery-to-artery embolism [23]. Transient ischemic attacks have been associated with intermittent compression, particularly of the ICA. Renard et al. [34] identified reduced distances between the SP, the HB, and the ICA as a significant risk factor for CA dissection. Yamaguchi et al. [41] explored the role of the perivascular mechanical environment in the site-specific pathogenesis of atherosclerosis, particularly within the CAs. However, findings from cross-sectional and longitudinal cohort studies indicate that a reduced hyoid–ICA distance does not necessarily correlate with an increased risk of atherosclerotic plaque formation or stenosis [42]. These insights underscore the complexity of vascular biomechanics and the need for further research to elucidate the precise role of mechanical forces in cerebrovascular pathology.

An additional perspective on CA dissection implicates the flip-flop phenomenon (FFP) as a potential mechanism underlying ischemic stroke [42]. The CAs are intricately positioned within the parapharyngeal space. They are surrounded by multiple anatomical structures, including bones, cartilage, and muscles, rendering them susceptible to mechanical influences induced by neck motion and swallowing. Notably, the HB and thyroid cartilage exhibit coordinated movement during swallowing, contributing to the CB dynamic displacement. Furthermore, the HB plays a pivotal role in speech, moving synchronously with the tongue [41]. Kinoshita et al. [42] have identified FFP—characterized by the Cas’ dynamic displacement due to interference from the HB during swallowing—as a factor associated with an elevated risk of ischemic stroke and symptomatic cerebrovascular events. These findings accentuate the necessity of considering biomechanical vascular interactions in stroke pathophysiology and highlight the importance of dynamic imaging modalities in high-risk individuals [42].

In instances of stroke with unknown etiology, especially when accompanied by cervical, pharyngeal, or otic pain, repetitive cervical movements, or prolonged cervical positioning, the possible displacement of the CAs by the HB necessitates a thorough investigation [11]. In these situations, angio-CT with dynamic head rotation may be a valuable diagnostic tool. Rapid head rotation heightens the risk of vascular injury, particularly on the contralateral side. The displacement of the ICA origin concerning the GH of the HB during head movements and swallowing in everyday activities may lead to chronic mechanical stimulation [11]. This repeated insult could trigger endothelial injury, thrombus formation, and cerebral artery embolism [41]. These findings underscore the need for a comprehensive vascular assessment in patients exhibiting these risk factors to promote early detection and intervention [41].

In addition to anticoagulation and antiplatelet therapy, management of this phenomenon may require surgical intervention, specifically the resection of the compressive structure, most commonly the GH [40]. Resection of the GH has shown effectiveness in alleviating neurological symptoms; however, postoperative complications such as dysphagia have been documented. Excessive resection may lead to significant functional impairments since the GH is an attachment site for essential muscle groups involved in swallowing [40]. Furthermore, a meticulous surgical technique prevents injury to adjacent vascular and neural structures [40]. Notably, iatrogenic damage to the superior thyroid artery and vein may result in cervical hematoma. In contrast, injury to the superior laryngeal nerve may present as vocal weakness, increased phonatory effort, and a sensation of tightness [40]. The necessity of this surgical approach continues to be a subject of debate. Liu et al. [38] advocate for endarterectomy as a preferable alternative, whereas Martinelli et al. [25] caution against endovascular intervention and stenting, citing unfavorable outcomes. These considerations highlight the necessity for individualized therapeutic strategies to optimize patient safety and clinical efficacy.

The complex topographical variants of the CAs significantly influence the surgical accessibility of the parapharyngeal space [3]. For example, a more lateral positioning of the ECA relative to the GH, observed in 18.5% of cases, may lead to complications during parapharyngeal surgical procedures. Surgeons should consider this anatomical variation when planning surgeries such as tonsillectomies or biopsies, as these anomalies could increase the risk of vascular injury. Notably, atypical trajectories of the CAs to the HB, where they may course medially to the GH, pose a potential risk for unsafe surgical approaches [11]. Existing literature underscores the propensity of these vascular structures to extend into this region, thereby complicating surgical interventions [11]. These variants are critical for otorhinolaryngological procedures, including tonsillectomy, peritonsillar abscess drainage, adenoidectomy, and pharyngeal lesion biopsies [11]. Furthermore, they warrant careful consideration during procedures such as biopsies or punctures of the pyriform sinus and should be recognized as a differential diagnosis in cases of pharyngeal wall bulging [8]. Given these variations, individualized preoperative imaging and assessment are paramount to mitigating iatrogenic complications and optimizing surgical outcomes.

It is imperative to recognize several limitations inherent in the present study. Although the sample size (*n* = 200 sides) is deemed acceptable, utilizing Manta’s classification system suggests that a larger sample could yield more comprehensive results. Furthermore, it is noteworthy that the participants were sourced from a specific geographical region, namely Athens, Greece. Lastly, given the retrospective and anatomical nature of this study, the patients’ medical histories were inadequately recorded as unknown. Therefore, future studies correlating these findings with clinical symptoms as well as with different imaging techniques, such as ultrasound and magnetic resonance imaging, would enhance our knowledge. Future studies should examine the impact of movements such as swallowing or head rotation on the carotid–hyoid relationship. Utilizing dynamic imaging in patients with chronic neck pain or dysphagia could assess whether repeated motions compress the ICA or ECA, which may potentially elevate stroke risk.

## 5. Conclusions

This study elucidates novel insights into the anatomical relationship (topography) between the CAs and the HB, emphasizing the critical clinical implications of these spatial associations. The analysis reveals that the ECA demonstrates the closest proximity to the GH of the HB, followed by the CCA and the ICA. Significantly, the GHL was discovered to correlate with the distance of these vessels inversely, reinforcing its role as a determinant of carotid–hyoid topography. In light of the clinical relevance of these anatomical relationships, the findings underscore the necessity of preoperative imaging to evaluate the potential proximity of the CAs to the HB, particularly in patients undergoing cervical procedures or presenting with unexplained cerebrovascular symptoms. Surgeons may utilize GHL and GHA measurements to evaluate the risk of arterial compression in imaging studies and subsequently adjust their surgical techniques to mitigate the potential for vascular injury. Although prior reports have suggested that these anatomical configurations may be associated with vascular compression syndromes, this study further elucidates the potential role of the HB as a mechanical stressor contributing to vascular pathology. Incorporating carotid–hyoid topography into preoperative CTA assessments may help identify patients at increased risk for vascular complications during head and neck surgeries.

## Figures and Tables

**Table 1:** The morphometric measurements of the greater horn (GH) of the hyoid bone (HB) according to sex and side. The results are presented as mean (standard deviation), GHL (greater horn length), and GHA (greater horn angle). Statistically significant results are highlighted with asterisk (*).

Mean Values of Parameters	Total (*n* = 200)	Male (*n* = 100)	Female (*n* = 100)	*p*-Value	Left (*n* = 100)	Right (*n* = 100)	*p*-Value
GHL	27.72 (3.91)	29.56 (3.67)	25.88 (3.24)	<0.001 *	27.54 (3.85)	27.90 (3.98)	0.604
GHA	110.36 (6.06)	109.62 (6.63)	111.09 (5.37)	0.134	111.07 (6.13)	109.64 (5.94)	0.116

**Table 2:** The correlation and regression analysis of the greater horn (GH) of the hyoid bone (HB) length (GHL) and greater horn angle (GHA) and the distances between the common carotid, external carotid, and internal carotid arteries (CCA, ECA, and ICA). Statistical significant results are highlighted with asterisk (*).

Parameters		R-Correlation	*p*-Value	B-Coefficients	*p*-Value
GH Morphometry	Distances (−)				
GHL	GH-CCA	−0.171	0.129	−0.077	0.336
GHA	GH-CCA	−0.100	0.376	−0.047	0.314
GHL	GH-ECA	−0.240	0.007 *	−0.252	<0.001 *
GHA	GH-ECA	+0.024	0.791	+0.013	0.787
GHL	GH-ICA	−0.233	0.008 *	−0.211	0.021 *
GHA	GH-ICA	+0.123	0.082	+0.142	0.091

## Data Availability

All the data are available upon reasonable request to the corresponding author (Maria Piagkou-mapian@med.uoa.gr).
